# Validating fPSA Glycoprofile as a Prostate Cancer Biomarker to Avoid Unnecessary Biopsies and Re-Biopsies

**DOI:** 10.3390/cancers12102988

**Published:** 2020-10-15

**Authors:** Tomas Bertok, Eduard Jane, Aniko Bertokova, Lenka Lorencova, Peter Zvara, Bozena Smolkova, Radek Kucera, Helmut Klocker, Jan Tkac

**Affiliations:** 1 Department of Glycobiotechnology, Institute of Chemistry, Slovak Academy of Sciences, Dubravska cesta 9, 845 38 Bratislava, Slovakia; Tomas.Bertok@savba.sk (T.B.); Eduard.Jane@savba.sk (E.J.); aniko.bertokova@savba.sk (A.B.); lenka.lorencova@savba.sk (L.L.); 2 Glycanostics, Ltd., Dubravska cesta 9, 845 38 Bratislava, Slovakia; 3 Department of Clinical Research, University of Southern Denmark, J. B. Winsløws Vej 23, 5000 Odense C, Denmark; pzvara@health.sdu.dk; 4 Department of Urology, Odense University Hospital, J. B. Winsløws Vej 4, 5000 Odense C, Denmark; 5 Department of Molecular Oncology, Cancer Research Institute, Biomedical Research Center of the Slovak Academy of Sciences, Dubravska cesta 9, 845 04 Bratislava, Slovakia; bozena.smolkova@savba.sk; 6 Department of Immunochemistry Diagnostics, University Hospital in Pilsen, E. Benese 1128/13, 301 00 Pilsen, Czech Republic; KUCERAR@fnplzen.cz; 7 Department of Urology, Medical University Innsbruck, Anichstrasse 35, A-6020 Innsbruck, Austria; helmut.klocker@i-med.ac.at

**Keywords:** prostate cancer, diagnostics, prognostics, glycans, fPSA, biomarkers

## Abstract

**Simple Summary:**

We have proved here that a magnetic bead-based assay using lectins can be effectively applied for glycoprofiling of free prostate specific antigen (fPSA). Such a glycan-based biomarker improves the detection of prostate cancer (PCa) in the PSA grey zone, the discrimination between clinically significant and insignificant cancer, and can significantly reduce the number of unnecessary prostate biopsies and re-biopsies, outperforming current second opinion tests such as percentage of fPSA (fPSA%) and the prostate health index (PHI).

**Abstract:**

Background: To compare the clinical performance of a new PCa serum biomarker based on fPSA glycoprofiling to fPSA% and PHI. Methods: Serum samples from men who underwent prostate biopsy due to increased PSA were used. A comparison between two equal groups (with histologically confirmed PCa or benign, non-cancer condition) was used for the clinical validation of a new glycan-based PCa oncomarker. SPSS and R software packages were used for the multiparametric analyses of the receiver operating curve (ROC) and for genetic algorithm metaheuristics. Results: When comparing the non-cancer and PCa cohorts, the combination of four fPSA glycoforms with two clinical parameters (PGI, prostate glycan index (PGI)) showed an area under receiver operating curve (AUC) value of 0.821 (95% CI 0.754–0.890). AUC values were 0.517 for PSA, 0.683 for fPSA%, and 0.737 for PHI. A glycan analysis was also applied to discriminate low-grade tumors (GS = 6) from significant tumors (GS ≥ 7). Conclusions: Compared to PSA on its own, or fPSA% and the PHI, PGI showed improved discrimination between presence and absence of PCa and in predicting clinically significant PCa. In addition, the use of PGI would help practitioners avoid 63.5% of unnecessary biopsies, while the use of fPSA% and PHI would help avoid 17.5% and 33.3% of biopsies, respectively, while missing four significant tumors (9.5%).

## 1. Introduction

Prostate cancer (PCa) is the second most frequent malignancy in men worldwide, counting 1,276,106 new cases and causing 358,989 deaths in 2018 [1]. PCa incidence is expected to increase to 2.1 million cases, with 633,328 annual deaths by 2035 [2,3]. The use of prostate-specific antigen (PSA) as a PCa biomarker is associated with a high false positive rate of up to 75% and a significant false negative rate (~15–17%). Thus, PSA contributes to unnecessary biopsies and overdiagnosis/overtreatment that significantly affect the quality of life and create a substantial social and economic burden [4,5].

Recently, new biomarkers such as prostate cancer antigen 3 (PCA3) or marker combinations such as the prostate health index (PHI) or the analysis of four kallikreins (4K) (intact PSA, human kallikrein 2, tPSA and fPSA) score, as well as multiparametric magnetic resonance imaging (mpMRI), have been introduced to improve the accuracy of detecting clinically significant PCa and to help in deciding whether a biopsy is needed [6,7,8]. A widely accepted standardized regimen for screening and early detection of PCa is yet to be established, and a need for further improvement using multi-analyte blood tests has been recognized [9,10].

PSA is a glycoprotein whose glycan significantly changes with PCa development/progression [4,5]. Glycosylation is a driver for cancer development/progression [11,12], and thus, glycoprofiling PSA could outperform currently used PCa tests [13]. Murphy et al. found that glycan analysis, in combination with other approaches (DNA analysis, transcriptomics, and proteomics), is a robust tool for tumor stratification [10].

Here we present a novel way for fPSA glycoprofiling: fPSA is captured on anti-fPSA antibody-coated magnetic beads with subsequent fPSA glycoprofiling. This is achieved by employing an ELISA-like format of analysis. Four lectins that recognize different glycan structures on fPSA were applied as sensitive markers to separate the non-cancer cohort from the PCa cohort. By profiling the archived serum samples of the men in both cohorts compared the accuracy of fPSA glycoprofiling to that of PSA, fPSA%, and the PHI in PCa diagnostics and prognostics.

## 2. Results

The technical validation of our assay was described in our patent application, where discrimination of healthy individuals over PCa patients using PSA glycoprofiling was successfully achieved [14].

### 2.1. fPSA Glycans and Their Analysis

Mass spectrometry combined with other techniques (Figures S1 and S2) and literature data [5] revealed a biantennary complex glycan as the most abundant glycan form on fPSA standard from healthy individuals (Figure S3A). A preferential glycan structure on fPSA from PCa patients was established based on our lectin-binding data and literature [4,5] (Figure S3B). Lectins *Aleuria aurantia* lectin (ALL), recognizing *gPSA1*, and *Sambucus nigra* agglutinin (SNA), binding *gPSA3*, strongly interacted with the fPSA standard from healthy individuals, while *Wisteria floribunda* lectin (WFL), recognizing *gPSA4*, and *Maackia amurensis* agglutinin (MAA), binding *gPSA2*, showed a weak interaction (Figure S2, Table S2).

### 2.2. Glycan Biomarkers Outperform Total PSA and fPSA% for PCa Detection

An analysis of the four glycan biomarkers in the 140 serum samples of men who presented with elevated serum PSA alongside clinical standard parameters (total PSA—tPSA, fPSA, fPSA%, and age) resulted in 1120 data points for which receiver operating characteristics (ROC) curves were constructed. Values for best biomarker combinations, including interactions (which are standardly calculated using software packages), are summarized in Table S3.

The combination of all clinical parameters and glycan biomarkers (Line 13, Table S3) offered an area under curve (AUC) value of 0.752 (95% CI 0.672–0.829). The same AUC value of 0.753 (95% CI 0.678–0.833) (Line 14, Table S3) was obtained for six markers when omitting fPSA and tPSA. This is why we investigated the remaining six markers in combination with their interactions (Table S3, Lines 17–24). The best combination of these six markers/parameters with interactions offered an AUC value of 0.821 (95% CI 0.754–0.890), a sensitivity of 64.3%, a specificity of 87.1%, and an accuracy of 75.7%, and is labeled as prostate glycan index (PGI, Line 17, Table S3).

**Figure 1:**
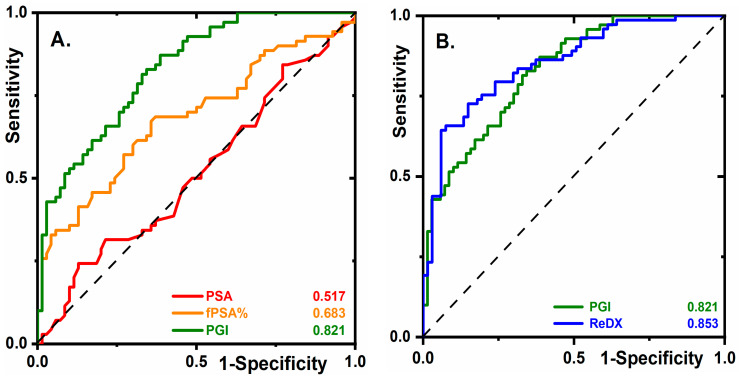
Diagnostic prostate cancer (PCa) biomarkers. (**A**) Receiver operating characteristics (ROC) curves for PSA, fPSA%, and prostate glycan index (PGI) (see Line 17, Table S3) are shown using 140 serum samples (70 samples from non-cancer cohort and 70 samples from PCa cohort). (**B**) ROC curves for PGI and for the case of 3 samples (2 samples with high-grade prostatic intraepithelial neoplasia (HG PIN) and 1 with PCa) being moved from the non-cancer cohort into the PCa cohort (labeled as “ReDX”). For more details, see the text.

A comparison of PGI’s clinical performance with that of tPSA or fPSA% illustrates the impressive superiority of PGI. At 95.0% sensitivity, PGI offered 45.7% specificity, an fPSA% specificity of 4.3%, and a tPSA specificity of 4.3% (Figure 1A). Thus at 95.0% sensitivity, alongside a low true negative rate (5.0%), there was a much lower false positive rate for PGI (54.3%) compared to fPSA% (95.7%).

### 2.3. Early Stage PCa Diagnostics

An analysis of non-cancer samples using PGI with a genetic algorithm (applied for re-classification of suspicious samples from the non-cancer cohort to the PCa cohort) revealed eight samples as suspicious. Re-checking identified two samples from high-grade prostatic intraepithelial neoplasia patients (HG PIN) closely related to PCa [15] and one sample from a PCa patient. In the remaining five suspicious samples, neither HG PIN nor PCa was confirmed. In Figure 1B, the ROC curve for PGI was compared with the ROC curve of re-diagnosis of two HG PIN samples and one PCa sample moved from the non-cancer cohort into the PCa cohort (ReDX). ReDX provided an AUC of 0.853, 95% CI 0.786–0.916 (64.3% sensitivity, 94.0% specificity, and 79.8% accuracy). Thus, PGI correctly identified the only “hidden” PCa case and the only two patients with HG PIN among the non-cancer cohort, a feature essential for early-stage PCa diagnostics.

### 2.4. The Diagnostic Performance of PGI Compared to PHI

**Figure 2:**
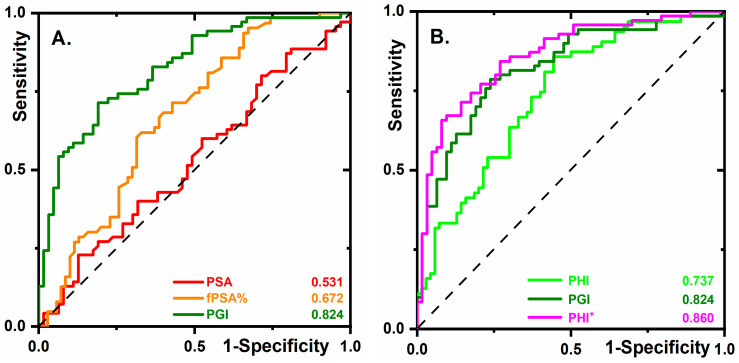
(**A**) ROC curves for the same biomarkers as shown in Figure 1A but using the 133 samples in total (63 samples from the non-cancer cohort and 70 samples from the PCa cohort). (**B**) ROC curves for PHI, PGI, and PHI+ (PHI combined with PGI), obtained by analysis of the 133 samples.

PHI is a formula that combines three PSA forms (tPSA, fPSA, and the PSA isoform [–2]proPSA) into a single score. It exhibits improved performance compared to either tPSA or fPSA% in determining the necessity of a biopsy [16]. PHI was measured in 133 serum samples, 70 samples in the PCa cohort, and 63 samples in the non-cancer cohort. The volume was insufficient for seven samples. The results indicate that the average ROC curve using the 133 samples did not change (Figure 2A) much compared to the analysis of the 140 samples (Figure 1A). Three approaches were compared in terms of clinical performance (Figure 2B) with the following AUC values: 0.737 (95% CI 0.648–0.815) for PHI, and 0.860 (95% CI 0.794–0.917) for PHI^+^ (PHI combined with PGI). At a specificity of 96.8%, the following sensitivities were obtained: 15.9% for PHI, 38.6% for PGI, and 48.6% for PHI^+^. Thus, PHI^+^ can significantly decrease the false negative rate.

### 2.5. PGI Decreases the Number of Unnecessary Biopsies

The clinical utility of PGI was compared to biomarkers applied to second opinion tests (i.e., fPSA% and PHI) by determining which men should be biopsied at 90% sensitivity, as previously proposed [7,17] (Figure 2A,B). The biomarker fPSA% avoided 11 out of 63 negative biopsies (17.5%), PHI avoided 21 biopsies (33.3%), and PGI avoided 40 out of 63 negative biopsies (63.5%). This means that, if applied as a second opinion test, PGI could have avoided a significant number (63.5%) of negative biopsies at 90% sensitivity, meaning that if the diagnoses were based solely on PGI, PCa would be missed in four cases. Compared to PHI, GPI avoided almost twice as many unnecessary negative biopsies.

### 2.6. The Prognostic Ability of Glycan Biomarkers in the Prediction of Low- and High-Grade Tumors

The potential of glycan-based biomarkers as prognostic PCa biomarkers for the discrimination of low grade (GS = 6) vs. significant tumors (GS ≥ 7) was examined. The best marker combination (Line 3, Table S4) provided an AUC of 0.632 (95% CI 0.496–0.768), which was higher compared to the PHI with an AUC of 0.568 (95% CI 0.430–0.705), and fPSA% with an AUC of 0.519 (95% CI 0.381–0.657). At a sensitivity of 90%, all three biomarkers missed only four significant PCa tumors (Table S4). When applying the glycan-based biomarker and the PHI, four PCa patients with GS 3 + 4 (14.8%) were missed, while the application of fPSA% resulted in two PCa patients with GS 3 + 4 (7.4%), one with GS 4 + 3 (33.3%), and one with GS 4 + 5 (25.0%) being missed. The number of avoided re-biopsies was much higher for glycan-based biomarkers (32.1%) compared to the PHI (0%), while maintaining the same number and grade of missed significant tumors. Thus, the glycan-based analysis is a useful approach in guiding a decision concerning the necessity of a re-biopsy or therapy/treatment.

## 3. Discussion

The alteration of the glycosylation pattern has a high potential for early tumor detection. Our results demonstrate the power of the glycan-based approach. The glycan pattern alone has a high discriminatory power for PCa detection, which can be further increased in combination with other parameters and the PHI. Furthermore, the magnetic bead-based assay is compatible with automatic assay formats developed by Roche, Beckman, and other companies.

The novelty of our study is in the analysis of four different glycans on fPSA using lectins. While all previous studies have considered a change in one glycan a PCa biomarker [18,19,20], the determination of four glycans on PSA allowed us to perform a multiparametric analysis, which significantly increased the AUC values in comparison with a single parametric analysis (Table S3).

Although several previously published studies have used PSA glycoprofiling as a diagnostic/prognostic PCa biomarker, those studies usually used human serum samples outside the grey zone [5,19,21]. Since the serum samples from the non-cancer control cohort and from PCa patients used in our study have a very similar PSA level (Table 1), the human serum samples applied in this study were extremely challenging for analysis. This statement can be highlighted by the fact that tPSA offered an AUC value of 0.517 (Table S3), which is much lower compared to the generally accepted AUC value of 0.68 [5]. Moreover, in Yoneyama’s work, tPSA offered an AUC of 0.61 when comparing a non-cancer cohort with a PCa cohort from a grey zone [20]. Even though the human serum samples applied in our study are extremely challenging for analysis, PGI offered an AUC of 0.853 (ReDX in Figure 1B). When serum samples from a grey zone were glycoprofiled in the literature, AUC values of 0.84 (95% CI 0.79–0.88) [20], or of 0.752 (95% CI 0.690–0.813) [19] were obtained.

The existing trend is to combine several variables into one test for PCa diagnostics in order to improve clinical performance. Three isoforms of PSA are needed for the PHI calculation [5]. Protein-based variables (four kallikreins) are combined with other variables (age, digital rectal examination (DRE) results, prior biopsy—yes, no) in order to calculate the 4K score [5]. In addition to the analysis of proteins, there are tests relying on the analysis of genetic markers such as PCA3 [22] and TMPRSS2:ERG [6]. The power of the serological multi-analyte/biomarker analysis for early tumor detection (CancerSEEK test) was established by the combined analysis of proteins and mutations in cell-free DNA [9]. A true potential for the integration of glycan-based assays to distinguish indolent localized PCa from aggressive non-localized PCa was revealed by the integration of six different types of biomarker blocks (clinical data, DNA methylation, coding and non-coding transcripts, proteins, and glycans) [10]. However, the glycan analysis was based on an instrumental-based approach [10], which is not compatible with an easy to perform ELISA format assay.

Recent guidelines for prostate biopsy recommend multiparametric magnetic resonance imaging (mpMRI) also for biopsy naïve patients. In this retrospective study, mpMRI data were not available for all subjects and were not considered, which is a limitation. In the current clinical setting for biopsy decision, the ability of the PSA glycoprofile to identify significant cancer could improve the accuracy of the preselection of men who have to undergo expensive imaging and subsequent biopsy, thus reducing costs and the number of negative biopsies without loss of significant tumor cases. The results presented here encourage prospective studies to test the positioning of the PGI prior to mpMRI in prostate biopsy decision making.

## 4. Materials and Methods

### 4.1. Clinical Cohorts

Archived serum samples from 140 males who participated in the Tyrolean prostate cancer early detection program of the Department of Urology, Medical University Innsbruck, Austria, between 2013 and 2015 were used. All men underwent prostate transrectal ultrasound-guided prostate biopsy after presenting with elevated serum PSA. The clinical characteristics of study participants are summarized in Table 1. The study was approved by the Ethics Committee of the Medical University Innsbruck, Austria (EK Nr: 1257/2017). Ten to fifteen transrectal prostate core biopsies (10 systematic, up to five targeted by contrast-enhanced color Doppler ultrasound and/or real-time sono-elastography) [23,24] were sampled from each patient and analyzed using standard histopathological procedures. Based on the biopsy results, a cancer and a non-cancer cohort was chosen to fulfill the criteria of a “grey zone” serum PSA [7] while being age and tPSA level matched (Table 1). The level of PSA in the samples was within the range of 2–10 ng/mL (except for 1 sample in the non-cancer cohort, tPSA = 10.7 ng/mL). On the basis of prostate biopsy tumor grades, the prostate cancer cohort was subdivided into a low grade (low-risk tumors, Gleason score 6) and significant (high-risk tumors, Gleason score ≥ 7) cancer sub-groups.

### 4.2. Glycoprofiling of fPSA, Data Analysis, and Statistics

A magnetic bead-based ELISA was developed to separate fPSA from samples using a magnetic field with a subsequent glycoprofiling of fPSA using lectins for the analysis of four glycans on fPSA as PCa biomarkers. Assay development was extensively characterized (Section S2 in Supporting Information file). fPSA glycoprofiling has the following beneficial features: (1) anti-fPSA was immobilized on magnetic beads (Micromod Partikeltechnologie GmbH, Rostock, Germany) to immunocapture fPSA from serum (Figure S4); (2) fPSA immunocomplex beads were applied to lectins that were immobilized on an ELISA plate (Sigma-Aldrich, Bratislava, Slovakia), so there was no need to release fPSA from the immunocomplex; (3) peroxidase conjugated to magnetic beads was used for an optical signal generation; (4) simplified assay procedure and a considerably reduced assay time compared to literature [25].

All computations were performed using R software (version 3.4.4) with Classification and Regression Training (CARET) and a GLM package [7,26]. The genetic algorithm under R was performed with the Genetic Algorithm (GA) package. Data were re-checked with SPSS software (IBM). Receiver operating characteristic (ROC) curves and area under the curve (AUC) parameter for individual markers, and their combinations were constructed in R software [27], using additional software packages [28]. All confidence intervals (CIs) presented are 95% two-sided bootstrap intervals.

## 5. Conclusions

To the best of our knowledge, this is the first comprehensive study showing the true potential of glycan-based analysis using lectins for the management of PCa patients (diagnostics and prognosis). A glycan-based analysis, combined with other biomarkers in ELISA assay formats, has higher sensitivity and specificity than both fPSA% and the PHI in detecting PCa and can reduce the number of unnecessary biopsies and re-biopsies. In addition, our data provide preliminary evidence of the power of this test to identify clinically significant prostate cancer. PGI correctly identified the only “hidden” PCa case and the only two patients with a pre-cancerous stage among the non-cancer cohort, a feature essential for early-stage PCa diagnostics. The approach applied here to fPSA glycoprofiling using anti-fPSA antibodies attached to magnetic particles is highly innovative, has several beneficial features, and is compatible with automatic machines developed by Beckman Coulter, Roche, etc.

## 6. Patents

The authors Tomas Bertok and Jan Tkac are inventors of PCT-patent application PCT/EP2019/057386 and have shares in Glycanostics Ltd. (Bratislava, Slovakia) being the applicant of said PCT-application.

## Figures and Tables

**Table 1:** Characteristics of cancer and non-cancer participants included in this study.

Characteristics	Participants, *n* = 140	
Biopsy Result	Non-Cancer 70 (50%)	Prostate Cancer 70 (50%)
**Age** **Average (range)** ≤60 year >60 year	60.2 *(49–77)* 37 (53%) 33 (47%)	64.1 *(40–79)* 27 (39%) 43 (61%)
**tPSA (ng/mL)** **Average (range)** ≤3 3–5 ≥5	5.4 *(2.5–10.7)* 6 (9%) 26 (37%) 38 (54%)	5.5 *(2.3–9.8)* 6 (9%) 26 (37%) 38 (54%)
**Prostate volume (mL)** ≤35 35–50 >50	13 (19%) 17 (24%) 40 (57%)	29 (41%) 21 (30%) 20 (29%)
**Biopsy results** Gleason score (patterns) 6 (3 + 3) 7 (3 + 4) 7 (4 + 3) 8 (3 + 5) 9 (4 + 5) 10 (5 + 5) Prostatitis Atrophy Benign hyperplasia High-grade PIN Previous biopsy *Follow-up carcinoma (ReDX)*	NA NA NA NA NA NA 43 (61%) 20 (29%) 28 (40%) 2 (3%) 11 (16%) *1*	28 (40%) 27 (39%) 3 (4%) 7 (10%) 4 (6%) 1 (1%) 12 (17%) 18 (26%) 6 (9%) 3 (4%) 3 (4%) NA
**Tumor risk groups** Gleason score		
Low grade GS = 6	NA	28 (40%)
Significant GS ≥ 7	NA	42 (60%)

NA: not applicable; ReDX: patient originally in the non-cancer cohort re-diagnosed as a PCa patient.

## Supplementary Materials

The following are available online at https://www.mdpi.com/2072-6694/12/10/2988/s1, Figure S1: MALDI-TOF MS/MS spectrum of permethylated N-glycans released from an fPSA molecule. One of the most abundant glycoforms (i.e., complex type sialylated biantennary N-glycan) present on the fPSA in healthy individuals is shown, Figure S2: Surface plasmon resonance (SPR) using a single-cycle kinetic analysis on a CM5 sensor chip modified with fPSA. Anti-fPSA antibody (black line) and chemically oxidized anti-fPSA antibody (red line) were investigated for binding to immobilized fPSA. The binding of four different lectins to immobilized fPSA is shown in the inset of the picture, Figure S3: (A) A typical glycan structure of an fPSA standard (from healthy individuals), as determined experimentally (see Supporting Information file). (B) One of the anticipated glycan structures of fPSA from PCa patients, drawn by taking into account literature data [5] and our experimental results. Abbreviations: Fuc: fucose; Man: mannose; Gal: galactose; Sia: sialic acid, i.e., *N*-acetylneuraminic acid; GlcNAc: *N*-acetylglucosamine; GalNAc: *N*-acetylgalactosamine; AAL: *Aleuria aurantia* lectin recognizing Fuc containing glycans, i.e., *gPSA1*; WFL: *Wisteria floribunda* lectin recognizing GlcNAc-GalNAc (LacdiNAc) glycans, i.e., *gPSA4*; MAA: *Maackia amurensis* agglutinin II recognizing α2,3-sialic acid-containing glycans, i.e., *gPSA2*; SNA: *Sambucus nigra* agglutinin I recognizing α2,6-sialic acid-linked glycans, i.e., *gPSA3*, Figure S4: Scanning electron microscopy (SEM) image of magnetic particles (*d* = 130 nm, dextran-coated) used for modification and subsequently for fPSA enrichment from real human serum samples. Unmodified particles, magnification 40,000× (left, inset magnification 50,000×) and modified particles after anti-fPSA antibody/HRP immobilization and surface blocking (right, magnification 40,000×) with a clearly distinguishable difference in nanoscale structure in case particles are enwrapped in a protein layer, Table S1: Lectins applied in the study with their glycan-binding preference, Table S2: Kinetic parameters obtained by single-cycle kinetics (kinetic titration) for anti-fPSA antibody, chemically oxidized anti-fPSA antibody and lectins AAL, MAA, SNA, and WFL using a sensor chip with immobilized fPSA, Table S3: Individual markers (1st section), their selected combinations (2nd section), selected combinations with interactions (3rd section) and corresponding sensitivities, specificities, accuracies, and AUC values obtained from ROC curves. In the 3rd section, tPSA and fPSA markers were deliberately omitted to lower the amount of significant markers entering the analysis down to six. These two markers did not have a significant impact on the analysis performance, Table S4: Number of missed cancers by Gleason score for glycan-based biomarkers, PHI and fPSA% (90% sensitivity). References [29,30,31,32,33,34,35,36] are cited in the Supplementary File.
