# Supplementary material for: Validating fPSA Glycoprofile as a Prostate Cancer Biomarker to Avoid Unnecessary Biopsies and Re-Biopsies
Source: Cancers (Basel). 2020 Oct 15;12(10):2988. doi: 10.3390/cancers12102988 (PMC7602627; doi:10.3390/cancers12102988)
Supplement: Supplementary file 1 [file cancers-12-02988-s001.pdf]

Supplementary Materials: Validating fPSA Glycoprofile as a Prostate Cancer Biomarker to Avoid Unnecessary Biopsies and Re-biopsies

Tomas Bertok, Eduard Jane, Aniko Bertokova, Lenka Lorencova, Peter Zvara, Bozena Smolkova, Radek Kucera, Helmut Klocker and Jan Tkac

S1. Materials and Methods

S1.1. Chemicals

All common chemicals (such as *N*-(3-dimethylaminopropyl)-*N'*-ethylcarbodiimide hydrochloride, *N*-hydroxysuccinimide, bovine serum albumin BSA, etc.) and buffer components were of p.a. grade and provided by Sigma Aldrich (St. Louis, USA). For all experiments, ultra-pure deionized water (G = 0.055 μS/cm) was used. Anti-fPSA antibody (ab10187) was purchased from Abcam (Cambridge, UK), biotinylated or unconjugated lectins from *Aleuria aurantia* (AAL), *Canavalia ensiformis* (Con A), *Maackia amurensis* agglutinin II (MAA), *Sambucus nigra* agglutinin I (SNA) and *Wisteria floribunda* (WFL) and Carbo-free blocking solution (10× concentrated) were purchased from Vector Labs (Burlingame, USA). Horseradish peroxidase (HRP, suitable for manufacturing of diagnostic kits) was obtained from Sigma Aldrich (St. Louis, USA). 130 nm magnetic dextran-coated particles (Nanomag®-D, 130 nm in diameter; MPs) were purchased from Micromod Partikeltechnologie GmbH (Rostock, Germany). Plates used for the assays were Nunc MaxiSorb™ (Sigma Aldrich, Bratislava, Slovakia) with high protein binding capacity (ThermoFischer, Waltham, USA). Zeba Spin desalting columns (7k MWCO) were purchased from ThermoFischer (Waltham, USA). Recombinant peptide-*N*-Glycosidase F (PNGase F) from *Flavobacterium meningosepticum* was purchased from Roche (Basel, Switzerland). Free-prostate specific antigen (fPSA, ≥ 95%) purified from a human seminal fluid was obtained from Fitzgerald Industries International (Acton, MA, USA). ENVI-Carb nonporous graphitized carbon (nPGC), 2,5-dihydroxybenzoic acid (DHB) as well as acetonitrile (ACN) for MS experiments were purchased from Sigma Aldrich (St. Louis, MO, USA). All buffers were freshly prepared and filtered using 0.2 μm sterile filters.

S1.2. Lectins

In this work five different lectins were employed in order to detect changes in the glycan profile of fPSA with their glycan binding structures shown in Table S1.

Table S1. Lectins applied in the study with their glycan binding preference.

Lectin	Source	Glycan Specificity
AAL	*Aleuria aurantia* mushrooms	fucose
ConA	*Canavalia ensiformis* bean seeds	mannose
MAA	*Maackia amurensis* seeds	α2,6-sialic acid
SNA	*Sambucus nigra* bark	α2,3-sialic acid
WFL	*Wisteria floribunda* seeds	GalNAc, GalNAc-GlcNAc (LacdiNAc)

Abbreviations: AAL: *Aleuria aurantia* lectin; ConA: Concanavalin A; MAA: *Maackia amurensis* agglutinin; SNA: *Sambucus nigra* agglutinin; WFL: *Wisteria floribunda* agglutinin; GalNAc: *N*-acetylgalactosamine; GlcNAc: *N*-acetylglucosamine.

S1.3. Oxidation and Control of Anti-fPSA´s Antibody Glycans

When combining antibodies and lectins in an assay format, it is important to prevent direct interaction of lectins with glycan part of an unoccupied antibody. This is why oxidation of glycans on antibodies is required in order to prevent assay interference.

Glycans of anti-fPSA antibodies were modified to prevent binding of lectins, which would interfere with glycoprofiling of fPSA. For that glycans of monoclonal anti-fPSA antibodies were oxidized

according to a previously published protocol [29,30] and blocked by a short amino acid. An enzyme-linked lectin binding assay (ELLBA) was used to control efficiency of oxidation of anti-fPSA antibody glycans. It was performed as previously described [31,32], with slight modifications. Shortly, wells of the ELISA plate were incubated with intact and chemically oxidized antibodies. The surface was subsequently blocked using a carbo-free blocking solution (CFBS; use of a 3% BSA solution yields the same results—data not shown) and let to interact with biotinylated lectins Con A (mannose-specific, strongly binding to glycans) and MAA (which should not bind to native IgG under normal circumstances anyway). After a short incubation with streptavidin-peroxidase (HRP) conjugate and OPD/H_2O_2 solution, signal was read at 490 nm after blank subtraction.

S1.4. Magnetic Enzyme-linked Lectin Binding Assay

MaxiSorb plates were modified by adding 100 µL of 50 µg/mL of lectin solutions to each well (4 different plates in total), shaken at RT for 1 h and incubated at 4 °C overnight. The plates were subsequently blocked using CFBS at RT by shaking and have been used immediately for the assay. Gradually thawed serum samples were diluted 5× by a phosphate buffer solution (100 mM, pH 7.4 PBS, filtered by a 0.22 µm sterile filter).

Modified magnetic particles (MPs/ox-Ab + HRP/CFBS) suspended in PBS were added to 5× diluted serum samples (at ratio of 5 + 1) to provide 10× diluted sera and incubated for 30 min at RT to capture fPSA. After that, MPs were washed and separated using a permanent magnet and diluted to an initial concentration of 5 mg/mL. These particles enriched with fPSA from human serum samples were applied on lectin-modified plates in a final volume of 100 µL and incubated for 20 min (RT, shaking). The procedure was continued with a washing step (200 µL of PBS), 100 µL of *o*-phenylenediamine (OPD, 1 mg/mL) solution in citrate-phosphate buffer (pH 4.6) with added 30% hydrogen peroxide (27 µL per 10 mL of buffer) to generated a signal after 10 min incubation (RT, dark). The reaction was stopped using 3.6 M H_2SO_4 and absorbance was read at 490 nm within 15 min.

All washing and pipetting steps for validation experiments with larger number of samples were performed by a multichannel pipette epMotion® 96 (Eppendorf, Hamburg, Germany) with a 2-position slider. fPSA standard was used for normalization of plate-to-plate variability and sample triplicates were used to estimate intra-batch variability for individual lectins, i.e. 7.0% for gPSA1, 7.4% for gPSA2, 8.2% for gPSA3 and 8.4% for gPSA4 (7.7% on average).Calibration curve offered a linear range from ~43 pg/mL to 4.3 ng/mL of fPSA for detection of gPSA4 (data not shown).

S1.5. Surface Plasmon Resonance

Successful modification of anti-fPSA antibody glycans by oxidation was controlled using surface plasmon resonance. All surface plasmon resonance (SPR) experiments were performed using Biacore X100 instrument (GE Healthcare, Tiefenbach, Austria). All procedures, i.e. pH immobilization scouting (using acetate buffers with pH 4, 4.5, 5 and 5.5), amine coupling (using EDC, NHS, ethanolamine) and single cycle kinetic (SCK) analyses (kinetic titrations for the estimation of kinetic parameters k_a, k_d and K_D) were performed using kits supplied by GE Healthcare, Austria and Biacore X100 control and evaluation software. Chips used for these experiments were CM5-carboxymethylated dextran-based sensor chips. After each cycle, the chip surface was regenerated using 50 mM NaOH.

S1.6. MALDI TOF/TOF Analysis of Released N-glycans

To establish the structure of fPSA glycans, glycans were split of the protein and analysed by MALDI TOF-TOF mass spectrometry. The fPSA sample (Fitzgerald, Crossville, USA) was processed according to previously published protocol [31]. Shortly, 10 µL fPSA sample (12 µg/mL) was mixed with 40 µl of 10 mM Tris pH 7.5 buffer + 0.1% SDS, heat denaturated (95 °C, 5 min) and cooled down to RT. Subsequently, 1 µL of PNGase F (1U/µL) was added and the sample was kept at 37 °C, overnight. Supelclean™ ENVI-Carb SPE column (Sigma Aldrich, St. Louis USA) was washed and 700 µL of 5% acetonitrile (ACN) per sample was added. Column was equilibrated with 5% ACN (1 mL) prior to sample enrichment, and then sample was applied to the column twice to bind glycans. Column was washed with 5% ACN (no glycan

release) and then glycans were eluted by 1 mL of a mixture (60% ACN + 0.1% TFA). Sample was lyophilized and stored at −80 °C.

To enhance the final MS signal, permethylation as a next step was performed. 7 grains of NaOH and silica-dried DMSO (1 mL) was mixed and subsequently CH_3I in 1 + 1 ratio was added to the mixture. After 45 min of shaking at RT (white solid is obtained at the end), the reaction was stopped by adding cold water (on ice) in three batches (3 × 100 µL) and finally vortexed. Reaction is exothermic—be careful! 500 µl of chloroform ($CHCl_3$) was added, sample was vortexed at 2000 rpm for 1 min, then centrifuged at 5000 rpm for 45 s. The upper phase (aqueous) was removed and pH was checked. The procedure was repeated until pH reached neutral value. A lower phase (chloroform-based) containing hydrophobic glycans was extracted. This last step was repeated at least 7×. A lower phase was then transferred to LoBind tubes and let to dry at 35 °C in order to remove water from the sample during last steps. Finally, the sample was mixed with DHB (matrix) in 50% methanol and then deposited (50 µL) on ground steel MALDI plates. The output from this experiment is shown in Figure S1.

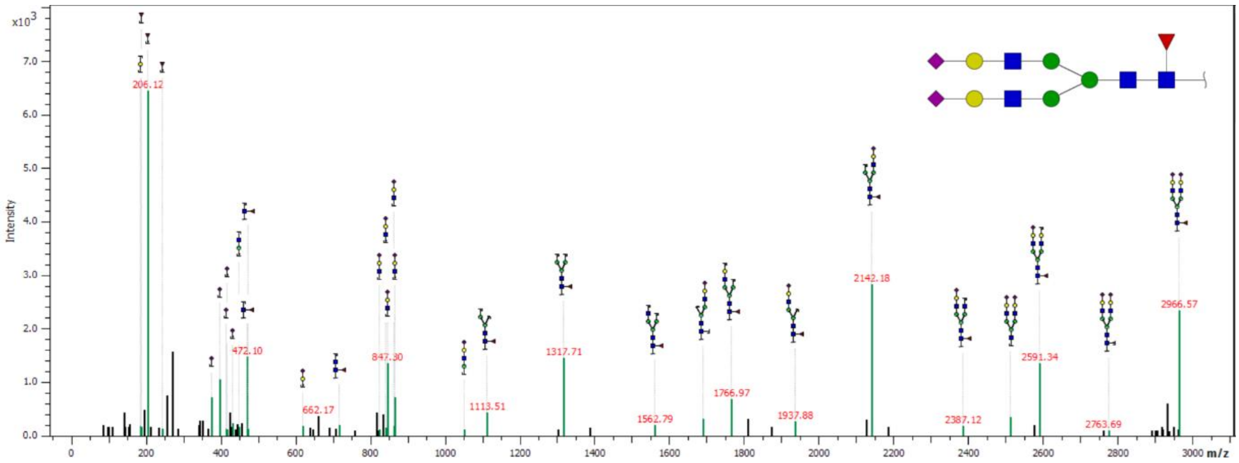

Figure S1. MALDI-TOF MS/MS spectrum of permethylated *N*-glycans released from a fPSA molecule. One of the most abundant glycoforms (*i. e.* complex type sialylated biantennary *N*-glycan) present on the fPSA in healthy individuals is shown.

S1.7. Magnetic Particles Characterization

Dextran-coated Nanomag® magnetic particles were employed to immobilize anti-fPSA antibodies and HRP. The beads were characterized before and after modification (using EDC/NHS chemistry—amine coupling) with anti-fPSA antibody/HRP mixture and subsequent blocking with CFBS using scanning electron microscopy (SEM) and dynamic light scattering (DLS). The particles of this size could easily be separated for washing steps with permanent neodymium magnets. Working concentration for these modified particles was 5 mg/mL.

S1.8. PHI Analysis

Serum levels of total PSA (tPSA), free PSA (fPSA) and [-2]proPSA were determined using the ACCESS chemiluminescent assays (Beckman Coulter, Brea, CA, USA). Subsequently, fPSA% and the PHI were calculated using the formulas: fPSA% = (fPSA/tPSA) × 100 and PHI = (−2proPSA/fPSA) × √tPSA.

S2. Results

S2.1. Control of Binding Properties of Glycan Oxidized anti-fPSA Antibodies

In order to control if modification of anti-fPSA antibody glycans was successful interactions of oxidized (oxAb) and non-oxidized antibodies (Ab) with lectins (Con A—a mannose binding, quite non-specific lectin binding most of the glycoproteins, and MAA, a specific lectin, which should not bind the IgG antibodies) were investigated using a previously described enzyme-linked lectin binding assay (ELLBA) [32,33]. The interactions of oxAb with lectins were significantly suppressed indicating a successful oxidation of anti-fPSA antibodies. Kinetic parameters of both antibodies (Ab and oxAb) were

compared using surface plasmon resonance on a CM5 chip with immobilized fPSA using a kinetic titration in a single cycle mode of analysis (Figure S1). The amount of fPSA immobilized on the chip surface was calculated according to the manufacturer´s recommendation (i.e. 1 R.U. = 1 pg mm^{-2}) as ~4.9 ng mm^{-2}. Other lectins involved in this study were analysed as well on the same chip interface after repeated regeneration.

After pH scouting, immobilization and single cycle kinetic analyses, 1:1 binding model was applied for the obtained data set. The results showed that oxAb preserved binding to its analyte—fPSA (Table S2 and Figure S1). Free PSA was immobilized on EDC/NHS activated CM5 sensor chip for 420 s under a flow rate of 30 μL/min from a stock solution (50 μl/mL) in an acetate buffer pH 4 (the best coupling buffer identified in a pre-concentration pH scouting).

Table S2. Kinetic parameters obtained by single cycle kinetics (kinetic titration) for anti-fPSA antibody, chemically oxidized anti-fPSA antibody and lectins AAL, MAA, SNA and WFL using a sensor chip with immobilized fPSA.

Parameters	*Ab*	*oxAb*	*AAL*	*MAA*	*SNA*	*WFL*
k_a (1/Ms)	2.72×10^4	0.15×10^4	3.40×10^4	0.31×10^4	3.63×10^4	0.59×10^4
k_d (1/s)	3.13×10^{-5}	7.65×10^{-4}	9.22×10^{-4}	1.16×10^{-4}	3.42×10^{-4}	5.65×10^{-4}
K_D^{app} (M)	1.15×10^{-9}	5.26×10^{-7}	2.71×10^{-8}	3.72×10^{-8}	9.40×10^{-9}	9.59×10^{-8}

Abbreviations: Ab: anti-fPSA antibody; oxAb: chemically oxidized anti-fPSA antibody; AAL: *Aleuria aurantia* lectin; MAA: *Maackia amurensis* agglutinin; SNA: *Sambucus nigra* agglutinin; WFL: *Wisteria floribunda* agglutinin.

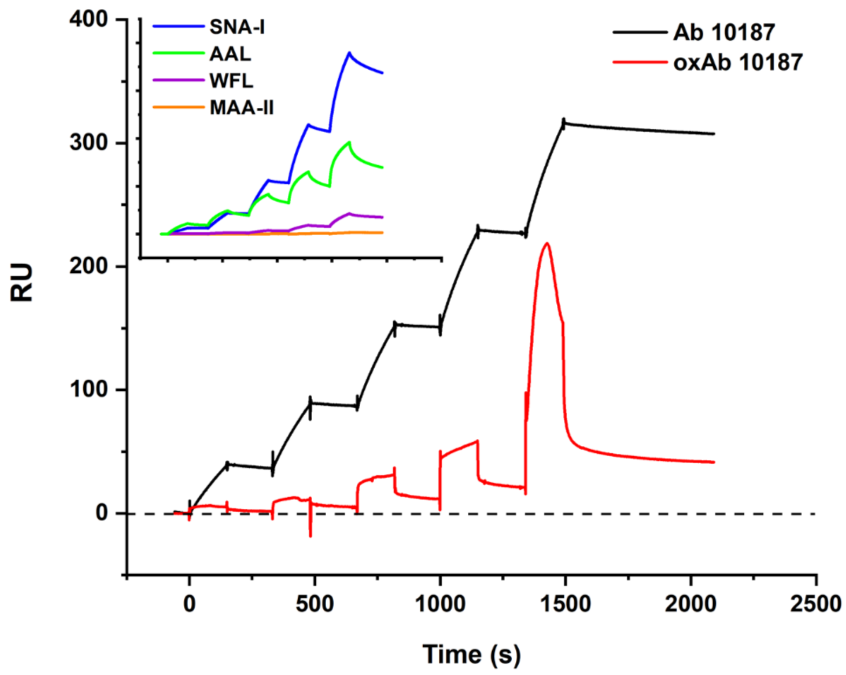

Figure S2. Surface plasmon resonance (SPR) using a single cycle kinetic analysis on CM5 sensor chip modified with fPSA. Anti-fPSA antibody (black line) and chemically oxidized anti-fPSA antibody (red line) were investigated for binding to immobilized fPSA. Binding of four different lectins to immobilized fPSA is shown in the inset of the picture.

S2.2. Processing and Analysis of MALDI TOF/TOF Data

All mass spectrometric measurements (MS) of derivatised *N*-glycans were performed on an UltrafleXtreme (BrukerDaltonics, MA, USA) in reflectron positive ion mode for MS and MS/MS (LIFT) analysis. The ions were recorded between 900 and 3,500 m/z and the laser intensity was optimized to give the best S/N ratio with the best maintenance of monoisotopic resolution for each sample. All acquired raw spectra were processed and analyzed by the FlexAnalysis and ProteinScape 3.0 software (Bruker

Daltonics, MA, USA). N-glycans, released from fPSA, were enriched by nPGC and analysed by MALDI TOF/TOF. Spectra were interpreted manually as well as confirmed by searching against the GlycomeDB tool of ProteinScape software.

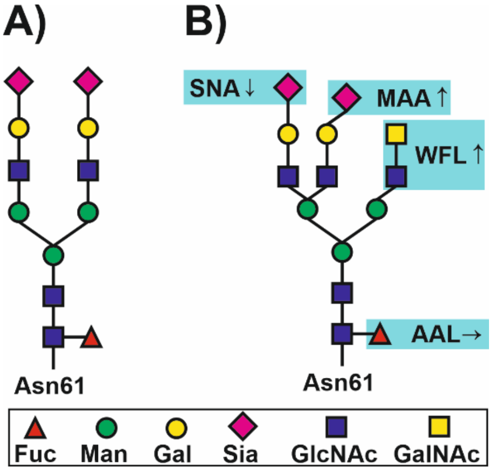

Figure S3: (**A**) A typical glycan structure of an fPSA standard (from healthy individuals), as determined experimentally (see Supporting information file). (**B**) One of the anticipated glycan structure of fPSA from PCa patients, drawn by taking into account literature data [5] and our experimental results. **Abbreviations**: Fuc: fucose; Man: mannose; Gal: galactose; Sia: sialic acid i.e. *N*-acetylneuraminic acid; GlcNAc: *N*-acetylglucosamine; GalNAc: *N*-acetylgalactosamine; AAL: *Aleuria aurantia* lectin recognizing Fuc containing glycans i.e. *gPSA1*; WFL: *Wisteria floribunda* lectin recognizing GlcNAc-GalNAc (LacdiNAc) glycans i.e. *gPSA4*; MAA: *Maackia amurensis* agglutinin II recognizing α2,3-sialic acid containing glycans i.e. *gPSA2*; SNA: *Sambucus nigra* agglutinin I recognizing α2,6-sialic acid-linked glycans i.e. *gPSA3*.

S2.3. Characterisation of Magnetic Particles and Their Characterization

Dextran coated magnetic particles (*d* = 130 nm, MPs; Micromod Partikeltechnologie GmbH, Rostock, Germany) had a hydrodynamic diameter determined by a dynamic light scattering (Zetasizer, Malvern Panalytical, Malvern, UK) as follows: (144 ± 6) nm for MPs, (154 ± 14) nm for MPs/ox-Ab + HRP and (183 ± 28) nm for MPs/ox-Ab + HRP/CFBS.

S2.4. Scanning Electron Microscopy

Scanning electron microscopy (SEM, Carl Zeiss EVO 40HV apparatus, Jena, Germany; after Au chemical vapour deposition treatment) was also used to observe the morphology changes after MPs modification and blocking (Figure S4), which surely led to increase in the hydrodynamic diameter of the modified MPs (because of a dense protein layer on the surface), as supported by the DLS data.

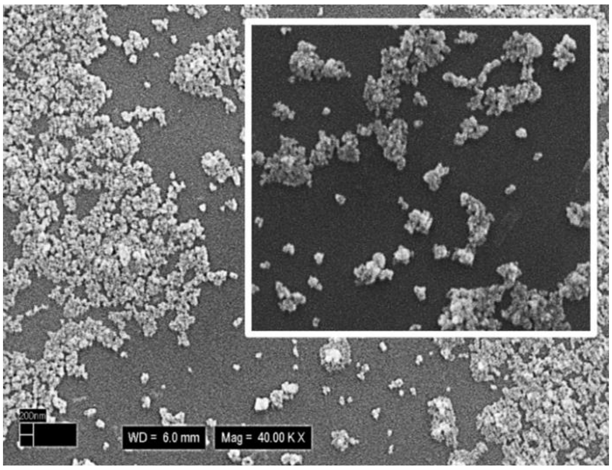

Figure S4. Scanning electron microscopy (SEM) image of magnetic particles (d = 130 nm, dextran-coated) used for modification and subsequently for fPSA enrichment from real human serum samples.

Unmodified particles, magnification 40,000× (left; inset magnification 50,000×) and modified particles after anti-fPSA antibody/HRP immobilization and surface blocking (right, magnification 40,000×) with clearly distinguishable difference in nanoscale structure in case particles are enwrapped in a protein layer.

S2.5. Evolutionary Algorithm and Patient Re-diagnostics (ReDx)

In the non-cancer cohort we wanted to find out if some of these samples can not be classified using our PGI as PCa samples. The data obtained for individual patients were re-evaluated using a genetic algorithm. Genetic algorithm (GA) is a metaheuristic method that mimics a biological process of natural selection and the most robust evolutionary algorithm available. This algorithm consists of three basic processes: natural selection, crossover and mutation [34]. Avoiding to find a local minima and integer optimization are the main advantage compared to the other type of optimizations [35]. Logistic regression as a standard method used for clinical data analysis is probabilistic and is thus more intuitive than its non-probabilistic counterparts.

Logistic regression for ReDx here was coupled with GA. The main reason was to find significant variables in dataset and the improvement of the selection process. Selection was set in difference of the AIC value (Akaike Information Criterion). In case of the GA/LogR (compared to the stepwise LogR) the fitness function can also be set differently [36].

$$\text{fitness(gen)} = \text{AUC} + \varrho - \varrho l/n \quad (1)$$

where AUC is area under curve, ρ is the compromise factor (set by the user), n is the total number of features, l is the number of selected features. In our case the method was modified to select patients in control group. The reason behind that was the fact that some patients could be misdiagnosed even after prostate biopsy or have some high risk factors for cancer.

The fitness function was set in R software. Note that patients were sorted on the health status. The non-cancer individuals were signed with 0 and cancer individuals were signed with 1. Variable gen is the vector of the zeros and ones (do not confuse this with the gen vector in R). Note that this algorithm in the package (GA) searches for the maximum (therefore negative sign before AIC value). If more than 15 patients should be re-categorized, the fitness function is penalized with the factor 1000. This penalization was done to hold the number of re-categorized patients low. Generation number was set on 10, and the population size was set on 100. The parents in crossover are selected via a stochastic uniform sampling. 80% of all samples in total were selected to crossover and the remaining ones were completed with mutation (10%) and elite individuals (10%). The mean value and the best value of the fitness function stalled after 3–5 generations. The algorithm was re-run 10 times and only 9 or 10 times selected patients were counted in.

Table S3. Individual markers (1st section), their selected combinations (2nd section), selected combinations with interactions (3rd section) and corresponding sensitivities, specificities, accuracies and AUC values obtained from ROC curves. In 3rd section, tPSA and fPSA markers were deliberately omitted to lower the amount of significant markers entering the analysis down to six. These two markers did not have a significant impact on the analysis performance.

.	Line.	Marker(s).	Spec (%).	Sens (%).	Acc (%).	AUC (95% CI)..
Individual.	1.	tPSA (ng/mL).	87.1.	24.3.	55.7.	0.517 (0.421 − 0.615)..
	2	fPSA (ng/mL)	57.1	71.4	64.3	0.649 (0.560 − 0.733)
	3	fPSA%	62.9	68.6	65.7	0.683 (0.596 − 0.770)
	4	Age	78.6	51.4	65.0	0.642 (0.550 − 0.731)
	5	gPSA1 = *g1*	75.7	45.7	60.7	0.509 (0.411 − 0.609)
	6	gPSA2 = *g2*	20.0	94.3	57.1	0.514 (0.389 − 0.583)
	7	gPSA3 = *g3*	48.6	72.9	60.7	0.575 (0.483 − 0.672)
	8	gPSA4 = g4	50.0	65.7	57.9	0.575 (0.483 − 0.670)
Combinations	9	fPSA% + fPSA + age + g4 + g3 + g2	88.6	51.4	70.0	0.751 (0.668 − 0.829)
	10	tPSA + fPSA% + fPSA + age + g4 + g3 + g1	77.1	61.4	69.3	0.751 (0.668 − 0.826)
	11	tPSA + fPSA% + fPSA + age + g4 + g1	75.7	62.9	69.3	0.752 (0.670 − 0.828)
	12	tPSA + fPSA + age + g4 + g3 + g2 + g1	72.9	68.6	70.7	0.752 (0.664 − 0.825)
	13	tPSA + fPSA% + fPSA + age + g4 + g3 + g2 + g1	78.6	61.4	70.0	0.752 (0.672 − 0.829)
	14	fPSA% + age + g4 + g3 + g2 + g1	74.3	67.1	70.7	0.753 (0.678 − 0.833)
	15	fPSA% + fPSA + age + g4 + g3 + g2 + g1	91.4	50.0	70.7	0.754 (0.672 − 0.829)
	16	tPSA + fPSA% + age + g4 + g3 + g2 + g1	75.7	64.3	70.0	0.755 (0.670 − 0.829)
Combinations and interactions	17	**PGI:** g4 + Age + age:fPSA% + age:g4 + fPSA%:g4:g3 + g4:g2 + g3:g1 + g2:g1	87.1	64.3	75.7	0.821 (0.754 − 0.890)
	18	fPSA% + age + age:fPSA% + age:g4 + g4:g3 + g4:g2 + g3:g1 + g2:g1	84.3	67.1	75.7	0.819 (0.747 − 0.884)
	19	Age + age:fPSA% + age:g4 + fPSA%:g3 + g4:g3 + g4:g2 + g3:g1 + g2:g1	88.6	60.0	74.3	0.818 (0.749 − 0.884)
	20	Age:fPSA% + fPSA%:g4 + g4:g3 + g4:g2 + g4:g1 + g3:g2 + g3:g1 + g2:g1	84.3	68.6	76.4	0.809 (0.734 − 0.877)
	21	fPSA% + age + age:fPSA% + g4:g3 + g4:g2 + g4:g1 + g3:g1 + g2:g1	90.0	57.1	73.6	0.808 (0.736 − 0.874)
	22	fPSA% + age:fPSA% + age:g1 + g4:g3 + g4:g2 + g4:g1 + g3:g1 + g2:g1	88.6	58.6	73.6	0.808 (0.730 − 0.876)
	23	fPSA% + age + age:fPSA% + age:g4 + age:g1 + g4:g1 + g3:g2 + g2:g1	78.6	74.3	76.4	0.807 (0.732 − 0.875)
	24	Age + age:fPSA% + fPSA%:g3 + g4:g3 + g4:g2 + g4:g1 + g3:g1 + g2:g1	94.3	52.9	73.6	0.807 (0.735 − 0.872)

Table S4. Number of missed cancers by Gleason score for glycan-based biomarkers, PHI and fPSA% (90% sensitivity).

Line	Marker(s)	3 + 4	4 + 3	3 + 5	4 + 4	5 + 3	4 + 5	5 + 5
1	fPSA%	2	1	0	0	0	1	0
2	PHI	4	0	0	0	0	0	0
3	fPSA% + age + fPSA + g4 + g3 + g2	4	0	0	0	0	0	0

© 2020 by the authors. Licensee MDPI, Basel, Switzerland. This article is an open access article distributed under the terms and conditions of the Creative Commons Attribution (CC BY) license (http://creativecommons.org/licenses/by/4.0/).
